# Cellular responses to targeted radionuclide therapy: rethinking radiobiology under continuous low dose rates

**DOI:** 10.3389/fonc.2026.1880443

**Published:** 2026-07-17

**Authors:** Pleun A.M. Engbers, Julie Nonnekens, Mariangela Sabatella

**Affiliations:** Erasmus MC Cancer Institute, University Medical Center Rotterdam, Department of Molecular Genetics, Department of Radiology and Nuclear Medicine, Rotterdam, Netherlands

**Keywords:** apoptosis, cell cycle, cellular response, DNA damage response, radiobiology, senescence, targeted radionuclide therapy

## Abstract

Targeted radionuclide therapy (TRT) delivers ionizing radiation directly to cancer cells through radio molecules that bind tumor-associated targets. Clinically successful examples include somatostatin receptor-directed TRT for neuroendocrine tumors, and prostate specific membrane antigen-targeted TRT for prostate cancer. Despite these advances, therapeutic efficacy remains limited by sublethal tumor doses, heterogeneous intratumoral uptake, and intrinsic radioresistance. A major challenge in improving TRT is the continued reliance on radiobiological concepts derived from external beam radiotherapy (EBRT). In contrast to EBRT, TRT is characterized by prolonged exposure times, low and variable dose rates, heterogeneous energy deposition, and radiation qualities ranging from low-linear energy transfer (LET) β^-^- particles to high-LET α-particles, resulting in distinct biological stress profiles that cannot be fully predicted from EBRT-based knowledge. This review summarizes the radiobiological properties that distinguish TRT from EBRT and describes how these features influence DNA damage induction and downstream cellular stress responses. Key cellular outcomes, including apoptosis, senescence, and alternatively regulated cell death pathways are discussed in relation to how dose rate kinetics and LET affect their timing and magnitude. By integrating concepts from radiation physics, DNA damage signaling, and cell fate mechanisms, this review highlights areas where mechanistic understanding remains limited and points to opportunities for refining TRT. A deeper understanding of these processes will support rational treatment design and help develop strategies that enhance tumor sensitivity while minimizing normal-tissue toxicity.

## 1 Introduction

Targeted radionuclide therapy (TRT) has emerged as a potent anti-cancer treatment in which radioactive isotopes are linked to tumor-targeting molecules. These molecules, typically labeled with β^-^- or α-particle emitters, are administered systemically and bind to tumor-specific entities, resulting in delivering of ionizing radiation (IR) directly to malignant cells (1). Clinically established examples include somatostatin receptor-targeted TRT using [^177^Lu]Lu-[DOTA-Tyr^3^]octreotate (^177^Lu-DOTA-TATE) for neuroendocrine tumors (2) and prostate specific membrane antigen (PSMA)-targeted TRT for prostate cancer (3). Despite significant clinical advancements, the efficacy of TRT remains restricted due to dose-limiting toxicities, insufficient targeting leading to sublethal tumor doses and the inherent radioresistance of certain malignancies (2, 4).

A major challenge in overcoming these limitations is the current reliance on radiobiological principles extrapolated from external beam radiotherapy (EBRT). While EBRT protocols have been optimized over decades to balance tumor control with tissue preservation, applying these directly to TRT is problematic due to fundamental differences in among others irradiation kinetics. EBRT typically delivers high dose rate radiation homogeneously over a short timeframe using an external source. In contrast, TRT delivers continuous, lower dose rate radiation internally over extended periods (5). For TRT, this results in simultaneous induction and repair of DNA damage, potentially altering the timing, accumulation, and processing of lesions in ways that cannot be inferred from EBRT. Since DNA double-strand breaks (DSBs) are the primary drivers of radiation-induced lethality, the distinct stress profile of TRT potentially provokes a differential biological response compared to EBRT (6). Moreover, TRT is characterized by heterogeneous dose distributions, dynamic changes in dose rate over time, and radiation qualities that range from low- linear energy transfer (LET) β^-^-particles to high-LET α-particles (7). These features not only influence spatial distribution and complexity of DNA damage, but may also shape the activation of intracellular signaling pathways and the selection of downstream cell fate programs. Consequently, there has been growing interest in advancing the understanding of the radiobiological effects induced by TRT to optimize its therapeutic efficacy (8, 9).

This review summarizes the radiobiological properties that distinguish TRT from EBRT and outlines how these properties shape the DNA damage response (DDR) and cellular stress responses. Importantly, the focus is placed on tumor-intrinsic responses, rather than emphasizing microenvironmental or systemic effect. In particular, we discuss the major cell fates triggered by TRT, including apoptosis, senescence, and alternatively regulated death pathways. Together, these insights clarify the current state of the art and highlight the key mechanistic gaps that must be addressed to better understand TRT-induced cellular responses.

## 2 Radiobiological properties

Cellular responses to irradiation depend strongly on how radiation is deposited within tissues and over time. These determinants can broadly be divided into three categories: 1) the physical characteristics of the radiation source; 2) the intrinsic radiosensitivity of the tissue or cells; and 3) the tumor microenvironment. In the following section, we will focus on the physical characteristics of the radiation source and discuss the radiobiological features that distinguish TRT from EBRT.

**Figure 1:**
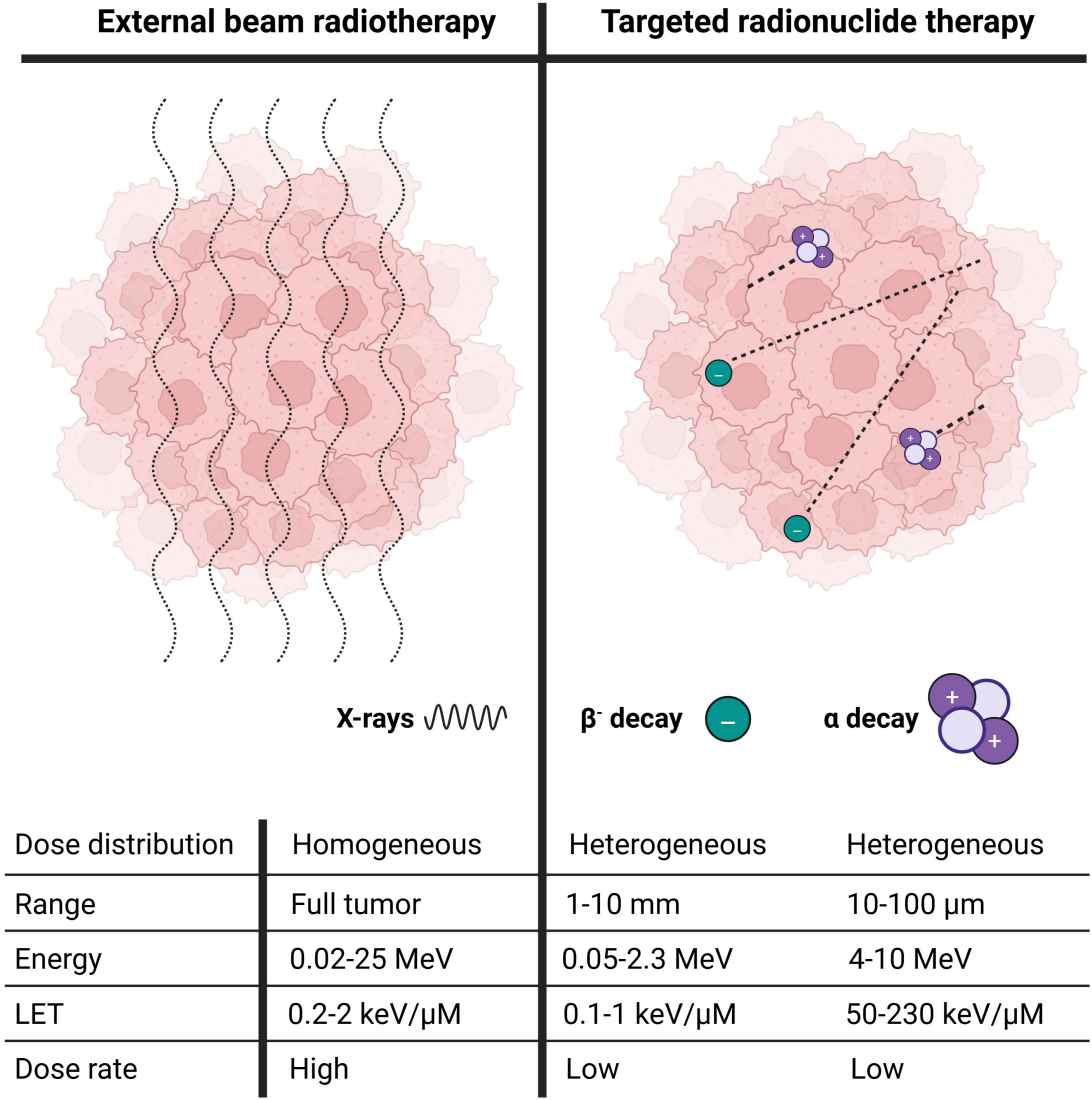
Physical characteristics of X-rays, β^-^-particles and α-particles. EBRT delivers low-LET X-rays uniformly across the tumor, whereas TRT produces heterogeneous irradiation from β^-^- and α-decays originating within tumor tissue. β^-^-particles provide millimeter-scale, low-LET cross-fire, while α-particles have short ranges and a high LET. Listed key physical parameters are adapted from (10, 146).

**Figure 2:**
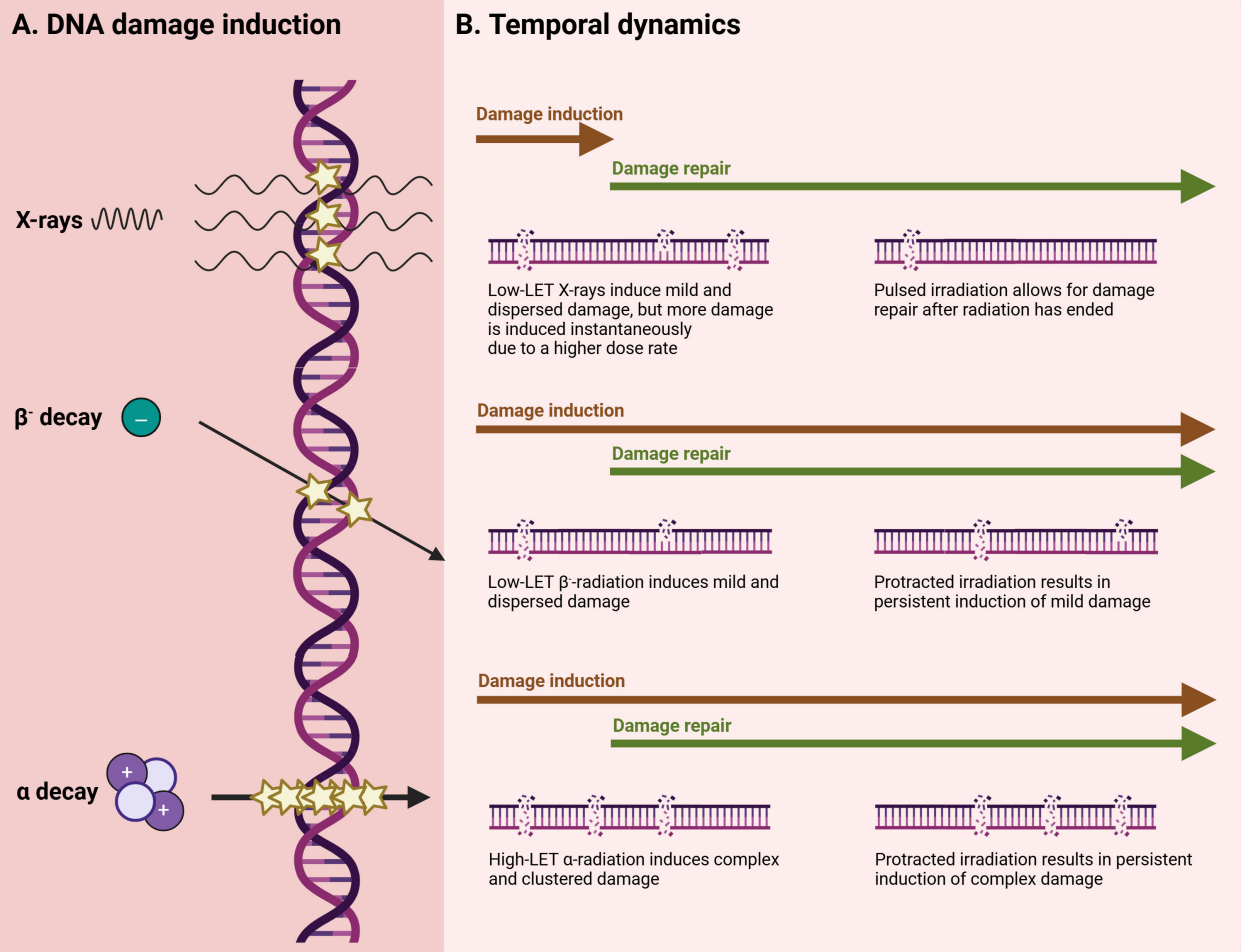
DNA damage induction and temporal dynamics of X-ray, β^-^- and α-irradiation. **(A)** Low-LET X-rays and β^-^-particles induce dispersed DNA damage, whereas high-LET α-particles generate dense ionization tracks and clustered DSBs. **(B)** Temporal dose-delivery patterns further modulate the balance between damage induction and repair. X-ray exposure induces damage in short, high dose rate pulses, allowing repair to occur after irradiation has ended. Low-LET β^-^-irradiation leads to gradual accumulation of mild damage under continuous low dose rate TRT. High-LET α-irradiation induces persistent complex damage during protracted exposure.

In EBRT, X-rays are administered externally and deposit energy relatively homogeneously across the tumor. These photons constitute low-LET radiation (~0.2 keV/μm), producing sparsely distributed ionization events and predominantly isolated DNA lesions (**Figures 1**, **2A**) (10). In TRT, the emitted radiation originates from radionuclides localized within or near tumor cells. Consequently, the spatial dose distribution is heterogeneous and depends on the extent, pattern and retention time of radiopharmaceutical uptake. β^-^-particles can travel over millimeter distances, enabling crossfire irradiation, and have low LET values (0.1–1 keV/μm) comparable to those of EBRT photons. In contrast, α-particles travel only tens of micrometers yet deposit high-LET energy (50–230 keV/μm), producing densely ionizing tracks and complex, spatially clustered DNA lesions, including multiple DSBs (DSB clusters), sometimes referred to as dual DSBs (**Figures 1**, **2A**) (10, 11). These clustered DSBs are more likely to be repaired incorrectly, leading to cytotoxic chromosomal aberrations. In addition, TRT-generated radiation tracks can also produce high-energy secondary (delta) electrons, which further extend the spatial distribution of ionization events and contribute to the formation of complex and clustered DNA damage (12).

Additionally, the dose rate and time of deposition strongly influences how DNA damage accumulates over time, as they determine the extent to which damage can be repaired between radiation events. Consequently, a substantial fraction of radiation-induced DNA damage is sublethal and repairable. Non-FLASH EBRT typically employs high dose rates, often reaching 1–24 Gy/min and the total absorbed dose is distributed in matter of seconds (13). In contrast, TRT delivers radiation continuously over hours to days, often at dose rates below 0.5 Gy/h (5). This protracted exposure allows cells to repair damage while new lesions are still being formed (**Figure 2B**). In classical radiobiology, this repairable damage component is described in the linear-quadratic (LQ) model as the β-term, representing lesions that can be repaired between radiation events but may become lethal when additional damage is induced before recovery (14). Under the low dose rate conditions of TRT, repair reduces the accumulation and interaction of sublethal lesions, thereby reducing the probability that they combine into cytotoxic events. At very low dose rates, a fraction of the delivered dose may instead counteract ongoing tumor cell proliferation rather than contribute to net cell killing, a phenomenon referred to as “wasted dose”, which depends in part on radionuclide half-life and dose rate kinetics (15).

This principle has been further illustrated by work showing that high dose rate administration of β^-^-emitter yttrium-90 results in significantly lower cell survival than delivery of the same total absorbed dose over the same exposure period but at a lower dose rate, indicating that rapid damage accumulation can overwhelm repair capacity (16). However, the relationship between dose rate and biological effect is non-linear. The “inverse dose rate effect” describes situations in which lower dose rates become more biologically effective than higher ones. This phenomenon has primarily been observed *in vitro*, often in rapidly proliferating cell lines, and may partly reflect experimental conditions or context-dependent cellular responses, analogous to or mechanistically related to low-dose hypersensitivity, for which comparable *in vivo* evidence remains limited. Its relevance therefore appears to depend strongly on tumor type and experimental context, and its underlying basis remains incompletely understood (17). Moreover, although low dose rates may initially produce stronger cytotoxicity, their cumulative impact often plateaus, ultimately resulting in a lower overall effect (18). This attenuation may arise from activation of compensatory or adaptive stress response pathways during prolonged irradiation. Fractionated administration of TRT therefore has been proposed as a strategy to circumvent these adaptive mechanisms and restore therapeutic effectiveness (19).

A further consideration is that the dose rate in TRT is inherently dynamic. It fluctuates over time due to variations in radiopharmaceutical uptake and clearance within the tumor, as well as radioactive decay (10). During the uptake phase, dose rate progressively increases as radionuclide accumulation rises. Thereafter, it declines during biological clearance and continues to decrease with physical decay. A faster initial increase in dose rate exerts a more pronounced cytotoxic effect than a gradual rise (20). This can be explained by slowly increasing dose rates allowing activation of adaptive radioprotective pathways that render tumor cells more resistant to subsequent irradiation (21).

These physical and temporal characteristics collectively shape the biological effect of TRT. A way to quantify this effect is through the biologically effective dose (BED), which described the biological impact of a treatment while accounting for both dose rate and the extent of repair that occurs during irradiation (22). BED is therefore useful for comparing the biological impact of treatments that deliver the same total absorbed dose but differ in how that dose is distributed over time. To compare biological effectiveness between different radiation modalities, the radiobiological effectiveness (RBE) is used. RBE quantifies the dose required to achieve a defined biological effect, typically cell death, relative to a reference radiation (usually EBRT), however its value depends strongly on the assay used and the timing of the biological readout. High-LET α-particles exhibit higher RBEs compared to both EBRT and β^-^-emitters, because their densely clustered DNA lesions overwhelm repair pathways at comparatively low doses (23–27). β^-^-emitters, despite having LET values similar to EBRT photons, often exhibit lower RBEs because their radiation is delivered at much lower dose rates, allowing repair to occur during exposure (18, 24, 28).

Although RBE is useful for comparing different radiation modalities, it does not fully capture the biological complexity of TRT. It does not account for the adaptive and dynamic responses that arise from continuous internal irradiation, nor does it reflect the heterogeneity of dose distribution within tumors. A deeper understanding of these biological processes, and of the mechanisms governing cellular death or survival following TRT, is therefore essential.

## 3 Cellular responses to TRT

### 3.1 Cell cycle

The cell cycle is a tightly regulated series of events that controls cellular growth, DNA replication, and division into two genetically identical daughter cells. It is classically segmented into four distinct phases: G1 (preparation for DNA synthesis), S (DNA replication), G2 (preparation for mitosis), and M (cell division). Progression through these phases is controlled by cyclins and cyclin-dependent kinases (CDKs). CDK4/6-Cyclin D and CDK2-Cyclin E complexes phosphorylate the retinoblastoma protein (RB) to facilitate S-phase entry, while CDK1-Cyclin A/B complexes drive the transition into mitosis. Beyond coordinating cell cycle transitions, these complexes function as critical control points that activate checkpoint mechanisms in response to cellular stress, including IR (29).

#### 3.1.1 Checkpoint activation and cell cycle arrest

**Figure 3:**
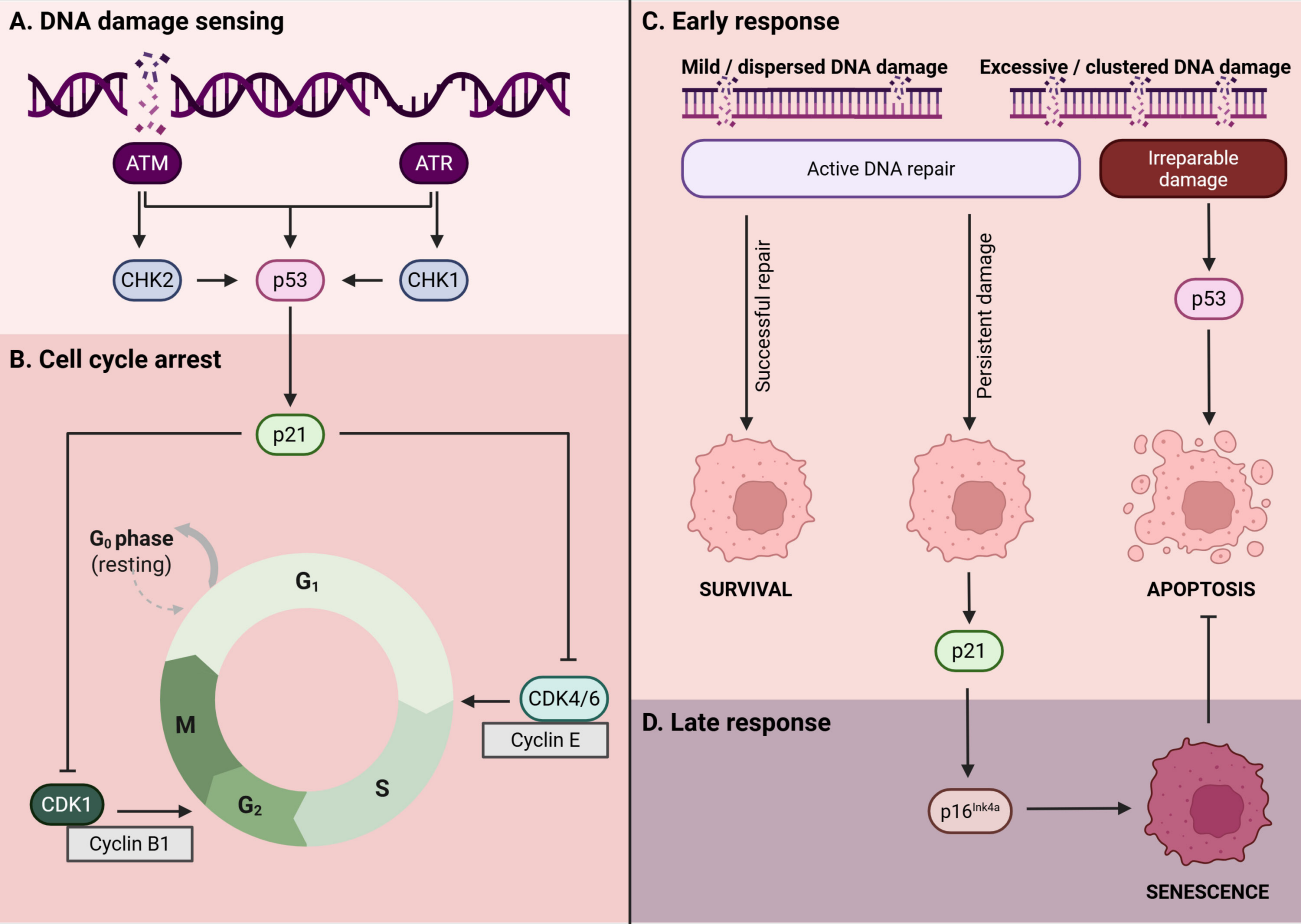
Induction of apoptotic and senescent response to radiation-induced DNA damage. **(A)** Radiation-induced DNA damage is detected by sensor kinases ATM and ATR. ATM primarily responds to DSBs, whereas ATR is activated by SSBs and replication stress. These kinases phosphorylate respectively checkpoint kinases CHK1 and CHK2, which stabilize and activate p53. **(B)** Activated p53 induces transcription of p21, which enforces G1 arrest via inhibition of CDK4/6 and contributes to G2 arrest through inhibition of CDK1. **(C)** During the early phase of cell cycle arrest, cells attempt damage repair. Successfully repaired lesions enable recovery and survival, whereas irreparable DNA damage triggers sustained p53 activation and apoptosis. **(D)** Cells that escape apoptosis but retain persistent DNA damage may enter a late response characterized by chronic p21 signaling and subsequent induction of p^16Inka^, ultimately leading to stable senescence, which is characteristically more resistant to apoptosis. ATM, ataxia-telangiectasia mutated; ATR, ataxia-telangiectasia and Rad3-related; CHK1, checkpoint kinase 1; CHK2, checkpoint kinase 2; p53, tumor protein p53; p21, cyclin-dependent kinase inhibitor 1; CDK1, cyclin-dependent kinase 1; CDK4/6, cyclin-dependent kinases 4 and 6; p16^Ink4a^, cyclin-dependent kinase inhibitor 2A.

Radiation-induced DNA damage is detected by the sensor kinases ataxia-telangiectasia mutated (ATM) and ataxia-telangiectasia and Rad3-related (ATR): ATM primarily responds to DSBs, whereas ATR is activated by single-strand breaks (SSBs) and replication stress (30). These kinases phosphorylate respectively the checkpoint kinase (CHK) 1 and CHK2, which stabilize and activate tumor protein p53 (p53) (**Figure 3A**). Nuclear accumulation of p53 initiates transcriptional programs that determine cellular fate (31). Early in the response, p53 induces cyclin-dependent kinase inhibitor 1 (p21), which enforces G1 arrest by inhibiting cyclin-dependent kinases (CDK) 4 and 6 (CDK4/6) and contributes to G2 arrest by inhibiting CDK1, thereby preventing Cyclin B1/CDK1-driven mitotic entry (**Figure 3B**) (30, 32). In addition to these transcriptional mechanisms, ATM- and ATR-dependent activation of CHK1/CHK2 rapidly inhibits cell division cycle 25 (CDC25) phosphatases, providing an immediate, transcription-independent block on CDK activity and allowing fast checkpoint engagement after irradiation (33). These checkpoints provide time for repair before replication and promote cell survival. However, when DNA damage exceeds repair capacity, p53 initiates the apoptotic response (**Figure 3C**) (34). Cells that evade apoptosis but retain unresolved damage may later enter senescence through persistent p21 signaling and upregulation of cyclin-dependent kinase inhibitor 2A (p16^Ink4a^) (35). Once senescence is established, cells become increasingly resistant to apoptotic cell death (**Figure 3D**) (36).

IR delivered as a pulsed EBRT exposure induces arrests in all major checkpoints, with the most pronounced accumulation occurring at the G2/M transition (37). A similar effect has been observed with somatostatin receptor-targeted TRT, in which lutetium-177 treatment induced a transient accumulation in G2/M 3 days after irradiation, returning to baseline by day 6 (38). In EBRT, this blockade occurs in two distinct waves (39). An early G2/M block appears within minutes after irradiation mediated by ATM, is largely dose-independent and reflects the inability of cells already in G2 to enter mitosis. In contrast, the later G2/M accumulation develops over several hours independent of ATM, reflecting cells irradiated in G1 or S that progress into G2 with unrepaired lesions. Although p53 is not required to initiate the G2/M arrest, both p53 and p21 are essential for its maintenance (40–42). This sustained G2 delay is thought to promote survival after sublethal DNA damage by allowing additional time for repair (43).

#### 3.1.2 Cell cycle dependent radiosensitivity and implications for TRT

Classical synchrony studies using EBRT have demonstrated that radiosensitivity varies across the cycle, with maximum sensitivity in G2/M and resistance in late S phase (44–46). This phase-dependence arises because pulsed irradiation delivers damage instantaneously, before cells can redistribute, making their position in the cycle an important determinant of outcome. These insights provided the rationale for strategies to enhance radiotherapy sensitivity by synchronizing cells with chemotherapeutic agents or CDK inhibitors, as well as through fractionated irradiation schedules (47).

In TRT, however, irradiation is delivered continuously over hours to days. Under these conditions, depending on the absorbed dose and dose rate, cells can traverse multiple phases during exposure, with potential repeated activation of checkpoints and redistribution across the cell cycle. As a result, the cell cycle position at treatment initiation may play a less prominent role than in EBRT. Nevertheless, the cell cycle can still influence TRT sensitivity, and its effects may differ between low- and high-LET radionuclides.

This was illustrated by a synchronization study comparing α-particle irradiation from astatine-211 with X-rays, which showed that RBE varied three- to eight-fold across different cell cycle phases (48). Unlike X-rays, which showed highest sensitivity during mitosis, α-particle irradiated cells were slightly more resistant in mitosis than in G1 or S phase. This was attributed to the highly compact chromatin architecture in mitosis, which reduces the likelihood that a short-range α-track will traverse DNA. On the other hand, a study using α-particle TRT showed that synchronization in G2/M phase enhanced TRT toxicity (49), consistent with the greater complexity and repair-refractory nature of high-LET-induced DNA damage. These findings indicate that α-particles retain some degree of cell cycle phase-dependence, but with a pattern distinct from EBRT.

For low-LET β^-^-particle TRT radiosensitivity is shaped less by the initial cell cycle and more by the ability of cells to sustain a G2/M arrest during the prolonged period of irradiation. This principle was demonstrated in a lutetium-177-based study, which showed that radioresistant cell lines accumulated in G2/M phase through WEE1-dependent inhibitory phosphorylation of CDK1, allowing additional time for repair and promoting survival. Radiosensitive cells, by contrast, failed to maintain this arrest and underwent apoptosis or mitotic death (50). Pharmacological inhibition of WEE1 with MK-1775 abrogated this checkpoint and sensitized resistant cells to lutetium-177, underscoring the central role of CDK1 regulation in β^-^-particle TRT response. Consistent with this, MK-1775 enhanced the cytotoxicity of when administered simultaneously or after incubation with the radioligand, but not when given beforehand (51), supporting the notion that in low-LET TRT, sustained checkpoint engagement during ongoing DNA damage induction is more important than the initial cell cycle phase.

Together, these findings suggest that TRT does not follow the classical phase-dependence observed in EBRT. Instead, the response appears to be governed by the interplay between dose rate, LET, and the capacity to sustain or abrogate G2/M arrest during prolonged irradiation. Although the precise contribution of cell cycle position to TRT sensitivity remains incompletely defined, current evidence points toward checkpoint dynamics rather than instantaneous phase as the dominant determinant.

### 3.2 DNA damage repair

After damage induction, cells activate DNA damage response pathways to detect and repair radiation-induced lesions. IR induces a broad spectrum of DNA damage, including base damages, single-strand breaks (SSBs), and DSBs (52). These lesions arise both through direct ionization of DNA and indirectly through the generation of reactive oxygen species (ROS) following radiolysis of intracellular water. Low-LET radiation relies more heavily on ROS-mediated damage, whereas high-LET radiation toxicity is more defined by direct targeting of the DNA (53). In addition to dispersed lesions, IR (high-LET in particular) can induce clustered DNA damage or dual DSBs, where multiple lesions occur within one or two helical turns. These complex clusters are more difficult to repair and can interfere with the activity of repair enzymes, contributing to the high cytotoxicity of high-LET TRT (54). The local chromatin environment further modulates both the formation and repair of radiation-induced damage. While DNA in more open euchromatin is generally more susceptible to both damage induction and repair, densely packed heterochromatin restricts accessibility and exhibits delayed repair kinetics. As a result, complex lesions arising within heterochromatin tend to persist longer and are more prone to misrepair, thereby contributing disproportionately to genomic instability (55).

#### 3.2.1 DNA damage repair pathways

Cells rely on several DNA repair pathways to restore genomic integrity following radiation exposure. Base lesions and SSBs are primarily repaired through the base excision repair (BER) pathway, in which lesion-specific DNA glycosylases remove damaged bases to generate abasic sites that are subsequently processed by AP endonucleases, DNA polymerase β, and DNA ligase (56). Efficient repair of SSBs is essential, as unrepaired SSBs can be converted into DSBs when encountered by replication forks during S-phase, greatly increasing cytotoxicity (57).

DSBs represent one of the most deleterious forms of DNA damage and are repaired mainly by non-homologous end joining (NHEJ) and homologous recombination (HR). Classical NHEJ operates throughout the cell cycle and begins with Ku70/80 binding to DNA ends, followed by recruitment of DNA-dependent protein kinase catalytic subunit (DNA-PKcs) to facilitate end processing and ligation. NHEJ is rapid but inherently error-prone and can introduce small insertions or deletions at the repair junction (58). The majority of low-LET induced DSBs are repaired within hours after radiation, however a subset persists. The proportion of these persistent breaks increases with rising LET, reflecting the greater prevalence of complex DSBs that are less compatible with efficient NHEJ (52).

HR is a high-fidelity pathway restricted to S and G2 phases, when a sister chromatid is available as a repair template. HR is initiated by resection of the DNA ends to produce replication protein A-bound single-stranded DNA. BReast CAncer (BRCA) proteins then promote RAD51 loading, allowing formation of a nucleoprotein filament that performs homology search and strand invasion (58). Because clustered or complex DSBs generated by high-LET radiation are often processed by extensive end resection, cells tend to rely more heavily on HR for their repair, while classical NHEJ becomes less effective (26, 59).

When neither classical NHEJ nor HR can be efficiently engaged, cells may employ alternative end joining (alt-EJ), a slower and highly error-prone backup pathway that relies on limited end resection and annealing of short microhomologies. Alt-EJ, mediated by factors such as PARP1, XRCC1, and polymerase θ, frequently generates larger deletions and chromosomal rearrangements, thereby contributing to genomic instability (60).

#### 3.2.2 Radiosensitization using DDR inhibitors

Because DNA damage is a major driver of radiation-induced cell death, and efficient repair counteracts therapeutic efficiency, inhibiting DNA repair pathways has long been explored as a strategy to enhance radiotherapy. In EBRT, inhibition of BER/SSB repair, NHEJ, or HR consistently increases cellular radiosensitivity, supporting the concept that repressing repair can increase the cytotoxic effects of radiation (61). Given their comparable LET values, EBRT and TRT engage many of the same DDR pathways, however the timing of this engagement is likely to differ because of the distinct dose rate and delivery profiles of both modalities. Such differences in delivery kinetics can influence the duration and magnitude of DDR signaling, meaning that combination therapies optimized for EBRT cannot be directly extrapolated to PRRT (62).

Nevertheless, the principle of radiosensitization through DDR inhibition has been shown for TRT. Inhibition of NHEJ, for example through DNA-PKcs, has been shown to sensitize tumor cells to TRT and enhance therapeutic response (63, 64). Targeting SSB repair can also be effective, as unrepaired SSBs are converted into DSBs during replication. This is illustrated by studies combining ^177^Lu-DOTA-TATE with PARP inhibitors, which report synergistic effects on DSB formation and tumor cell killing (38, 65, 66). A major challenges of combining IR with DDR inhibition is their overlapping toxicity profiles, which can increase damage to healthy tissues (67). However, the protracted nature of TRT provides opportunities to adjust dosing schedule to maximize tumor-cell susceptibility while limiting healthy tissues toxicity.

Ultimately, the degree of radiosensitization achieved with DDR inhibitors depends also on how cells process accumulated DNA damage through downstream signaling pathways, which determine survival, arrest, or cell death.

### 3.3 Apoptosis

IR-induced cell death can proceed through several pathways, of which apoptosis is the most extensively characterized. Apoptosis is a tightly regulated form of programmed cell death that requires adenosine triphosphate (ATP) and serves primarily to eliminate damaged or pre-neoplastic cells, thereby preventing propagation of genomic instability. Apoptosis can manifest in two distinct temporal patterns: rapid- and delayed apoptosis. Rapid apoptosis refers to cell death before mitotic entry and occurs particularly in radiosensitive cell types such as leukocytes and intestinal crypt cells (68). Most cancer cells, however, undergo delayed apoptosis. This delayed form frequently follows mitotic catastrophe, in which cells enter mitosis with unrepaired DNA damage, resulting in chromosome segregation and structural abnormalities that ultimately trigger apoptotic collapse (69). Morphologically, apoptotic cells exhibit shrinkage, pyknosis, chromatin condensation, and membrane blebbing (70). These blebs, together with fragmented nuclear structures, form apoptotic bodies that are rapidly phagocytosed by neighboring cells or macrophages. Throughout this process, plasma membrane integrity is maintained, preventing inflammatory activation (71).

#### 3.3.1 Mechanistic pathways of apoptosis

**Figure 4:**
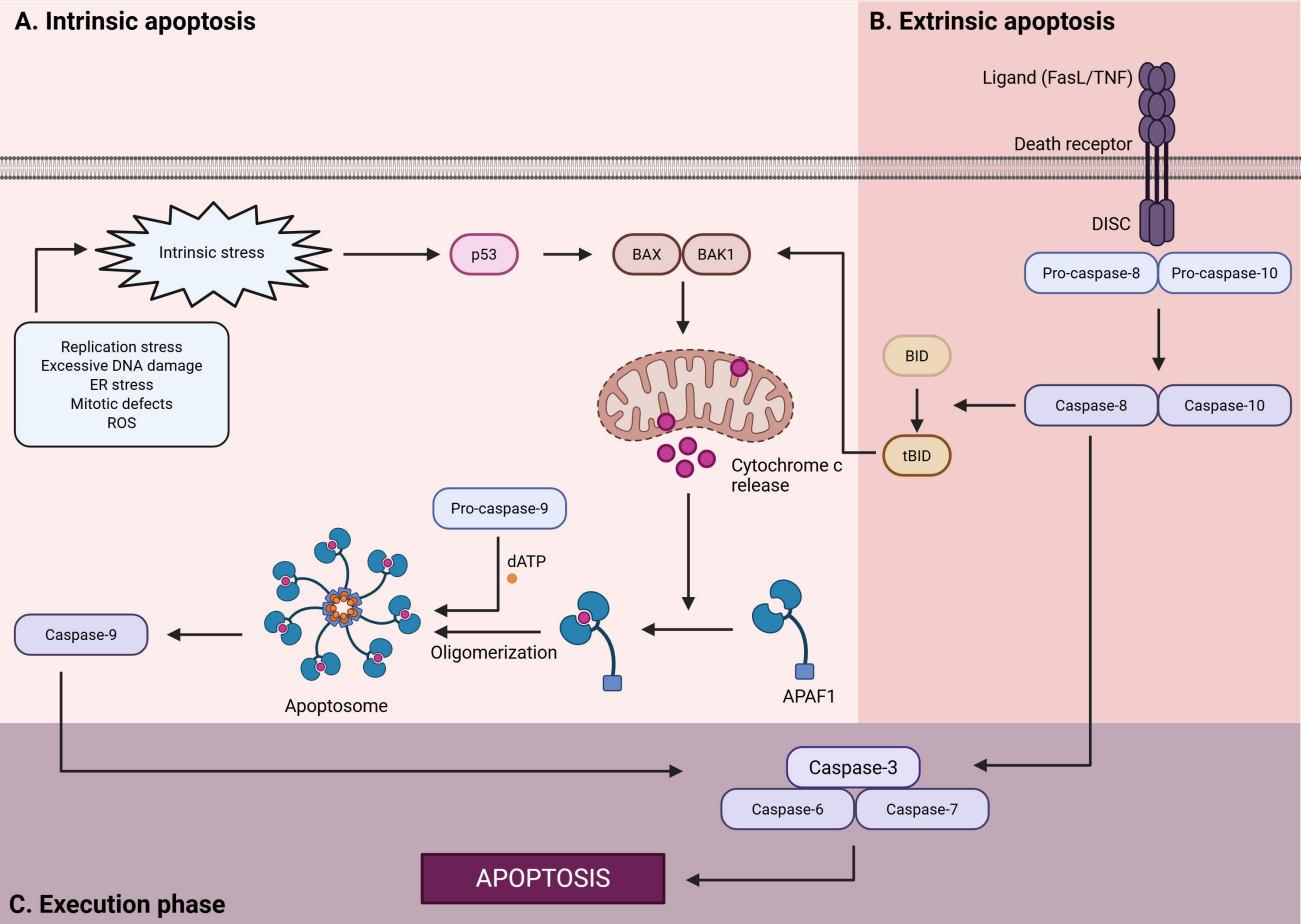
Extrinsic and intrinsic apoptosis pathways. **(A)** Intrinsic apoptosis is triggered by p53 activation in response to intracellular stressors. Activated p53 promotes pro-apoptotic BCL-2 family signaling, driving BAX/BAK1 oligomerization, mitochondrial outer-membrane permeabilization, and cytochrome c release. Cytochrome c forms the apoptosome with APAF1, dATP and pro-caspase-9, resulting in caspase-9 activation. **(B)** Extrinsic apoptosis is initiated by ligand binding to death receptors, leading to formation of the DISC and activation of caspases-8 and -10. Caspase-8 can also cleave BID to amplify mitochondrial permeabilization and engage the intrinsic pathway. **(C)** Both pathways converge on activation of effector caspase-3, supported by caspases-6 and -7, which execute apoptosis through chromatin condensation, cytoskeletal remodeling, and apoptotic body formation. ER, endoplasmic reticulum; ROS, reactive oxygen species; p53, tumor protein p53; BAK, BCL-2 antagonist/killer 1; BAX, BCL-2-associated X protein; BID, BH3-interacting domain death agonist; tBID, truncated-BID; APAF1, apoptotic peptidase activating factor 1; dATP, deoxyadenosine triphosphate.

Apoptosis can be initiated through intrinsic or extrinsic pathways, which converge on a shared execution phase. Intrinsic apoptosis is activated by intracellular stressors such as replication stress, excessive DNA damage, ROS, endoplasmic reticulum stress, or mitotic defects (72). This pathway is regulated by the B-cell lymphoma 2 (BCL-2) protein family. The pro-apoptotic proteins BCL2 associated X apoptosis regulator (BAX) and BCL2 antagonist/killer 1 (BAK1), both transcriptionally regulated by p53, oligomerize to form pores in the mitochondrial membrane, enabling cytochrome c release (73). Cytochrome c then associates with apoptotic peptidase activating factor 1 (APAF1), deoxyadenosine triphosphate (dATP), and pro-caspase-9 to form the apoptosome, which activates caspase-9 (**Figure 4A**) (74).

Extrinsic apoptosis is initiated by ligand (e.g. FasL/TNF) binding to cell-surface death receptors such as Fas (CD95/APO-1) or members of the TNF receptor superfamily. Ligand engagement triggers assembly of the death-inducing signaling complex (DISC), leading to activation of caspase-8 and in certain cell types, caspase-10 (75). Activated caspase-8 can in turn amplify intrinsic apoptotic signaling by cleaving pro-apoptotic protein BH3-interacting domain death agonist (BID) into truncated-BID (tBID), which promotes mitochondrial permeabilization and cytochrome c release, thereby facilitating caspase-9 activation (**Figure 4B**) (76).

Both pathways converge on the activation of effector caspase-3 which, supported by caspases-6 and -7, orchestrates the execution of apoptosis (**Figure 4C**). This final phase is characterized by the hallmark morphological changes described above (70).

#### 3.3.2 Apoptotic responses in TRT

While the pathways described above define the canonical execution of apoptosis, their activation and timing in the context of TRT is influenced by the radiation quality and delivery kinetics. High-LET α-emitters generate dense, clustered DSBs that rapidly overwhelm repair mechanisms, often leading to earlier onset of apoptosis compared with low-LET emitters when effects are considered at equivalent absorbed doses. For instance, treatment with α-emitter [^213^Bi]Bi-[DOTA-Tyr^3^]octreotide (^213^Bi-DOTATOC) has been reported to induce approximately fourfold higher levels of apoptosis than [^177^Lu]Lu-[DOTA-Tyr^3^]octreotide (^177^Lu-DOTATOC), with apoptotic activation detectable as early as 48 hours after start of treatment (25). Importantly, these comparisons cannot be based on administered activity alone, as α-emitters require far lower activities than β^-^-emitters to achieve comparable absorbed doses or biological effects. Consequently, apoptosis signaling following β^-^-emitter-based TRT typically peaks later, around 72–96 hours after treatment initiation (25, 38, 77–81). This delayed onset reflects multiple attempted repair cycles before the apoptotic threshold is reached and is characteristic of the low dose rate exposure inherent to β^-^-emitter-based TRT. Supporting this distinction, acute 2 Gy X-ray irradiation induces apoptosis more efficiently than ^177^Lu-DOTA-TATE, even when the latter delivers a twofold higher accumulated absorbed dose, highlighting the importance of delivery kinetics (28).

#### 3.3.3 Regulation by p53 in relation to dose scheduling

The transition from DNA damage sensing to apoptotic execution is primarily governed by p53, which integrates stress signals and determines cell fate (31). In TRT, the persistent nature of irradiation requires sustained p53 activation to reach the threshold for programmed death. Different studies have confirmed that stabilizing p53 or inhibiting its negative regulator MDM2 significantly enhances cellular sensitivity to ^177^Lu-DOTA-TATE, highlighting the importance of this pathway (82, 83). These observations suggest that therapeutic strategies aimed at reactivating or sustaining p53 activity, including MDM2 inhibition, may extend the window of apoptosis induction during TRT and thereby enhance treatment efficacy. *In vivo* profiling of GOT1 neuroendocrine tumor models show a temporal activation pattern, with p53 and its downstream effectors BAX and APOE peaking around 72 hours post-treatment (78–80). However, this activation is transient: by day 7, coinciding with early signs of tumor regrowth, p53 activity declines, and by day 41, gene expression shifts toward suppression of intrinsic apoptosis (79). This suggests a limited apoptotic window that can be overridden by pro-survival signaling.

The transient nature of p53 activation provides a rationale for optimizing TRT delivery, for example by fractionated administration. In a neuroendocrine GOT1 mouse model, division of a total activity of ^177^Lu-DOTA-TATE into multiple smaller administrations has been shown to induce delayed regrowth and prolonged overall survival compared to when administered as a single dose (19). Similarly, priming with 5 MBq ^177^Lu-DOTA-TATE followed by a second injection of 10 MBq enhanced apoptosis by prolonging p53 activation for up to 7 days and preventing late induction of anti-apoptotic genes, compared to a single injection of 10 MBq, where p53 activation is only observed at 3 days after treatment (78). Notably, priming doses can also alter the dominant apoptotic pathway: whereas single-dose ^177^Lu-DOTA-TATE activates only intrinsic apoptosis, priming-dose regimens engage both intrinsic and extrinsic pathways (78).

Importantly, apoptosis can proceed independently of p53. For example, leukemia cells lacking functional p53 can still activate caspase-3 in response to β^-^- and γ-radiation, indicating that p53-independent apoptotic routes, such as caspase-8-mediated mitochondrial amplification or stress-kinase pathways can compensate in certain contexts (84). This underscores that although p53 is a central regulator of radiation-induced apoptosis, it is not strictly required for cell death to occur. Because many tumors lack functional p53, it is essential to understand how TRT activates p53-independent death pathways. p53 status not only influences apoptotic competence but also affects shifts toward senescence or mitotic death (31), making it a key determinant of TRT response. Clarifying both p53-dependent and p53-independent mechanisms is therefore critical for optimizing TRT across diverse tumor genotypes.

#### 3.3.4 Extranuclear pathways contributing to apoptosis

While the DNA-centric model of radiation damage remains dominant, TRT can also activate apoptosis through membrane-associated mechanisms. α-particle and Auger-electron TRT specifically targeting the cell membrane induce ceramide production, a sphingolipid vital for membrane integrity (85, 86). Elevated ceramide levels promote aggregation of lipid rafts into signaling platforms that reorganize receptors and facilitate stress responses. These platforms can activate caspase-dependent pathways, p38 MAPK- and JNK signaling, and can also engage extrinsic apoptosis (87–90). Consistent with this, α-particle TRT-induced cell death was shown to involve activation of p38 and JNK (85). β^-^-radiation can likewise stimulate extrinsic apoptosis through upregulation of both the CD95/Fas receptor and ligand, enabling death-receptor activation (84).

### 3.4 Senescence

When apoptotic signaling does not reach the required threshold due to insufficient dose rate, persistent but sublethal DNA damage, or intrinsic resistance, cells may instead enter radiation-induced senescence. This process is a stable form of cell cycle arrest in which cells remain metabolically active but lose proliferative capacity. The transition between apoptosis and senescence is governed by multiple factors, including the extent and complexity of DNA damage, p53 signaling dynamics, repair capacity, chromatin context, and cell cycle phase, and is discussed in detail elsewhere (91). Senescence commonly arises when DNA damage is sustained but not immediately lethal, and is generally initiated in the G1 phase following activation of DNA damage checkpoints, although cells may transiently arrest in G2 before entering senescence upon returning to G1 (92). The primary biological function of senescence is to prevent propagation of cells carrying DNA damage. Accordingly, the accumulation of DNA lesions serves as the main trigger for senescence induction, with its onset largely determined by the nature and complexity of the damage (93). Senescence can be classified into distinct subtypes depending on the inducing stimulus. Replicative senescence arises from telomere shortening and is predominantly associated with aging. Oncogene-induced senescence is triggered by aberrant activation of specific oncogenes. Therapy-induced senescence arises when cytotoxic treatments, such as chemotherapy, EBRT and TRT, impose persistent DNA damage that arrests proliferation in cells that do not undergo apoptosis. Although these forms differ in their initiating signals, they converge on shared molecular pathways that enforce stable growth arrest (92).

#### 3.4.1 Mechanistic pathways of senescence

Senescent cells exhibit characteristic morphological and molecular features. Morphologically, they display cellular hypertrophy, a flattened shape, and formation of dense heterochromatin foci. Molecularly, senescence is associated with downregulation of cell cycle related genes, increased expression of senescence-associated β-galactosidase (SA- β-gal), and acquisition of a senescence-associated secretory phenotype (SASP), consisting of growth factors, pro-inflammatory cytokines, chemokines, proteases, and extracellular matrix components (94). The SASP is known to exert both tumor-suppressive and tumor-promoting effects: while it can reinforce growth arrest and limit uncontrolled proliferation, prolonged secretion of inflammatory cytokines and growth factors may remodel the microenvironment in ways that support tumor progression over time (92). The long-term persistence of senescent cells may further sustain these effects, contributing to chronic inflammation, tissue dysfunction, and potentially facilitating tumor recurrence (95).

**Figure 5:**
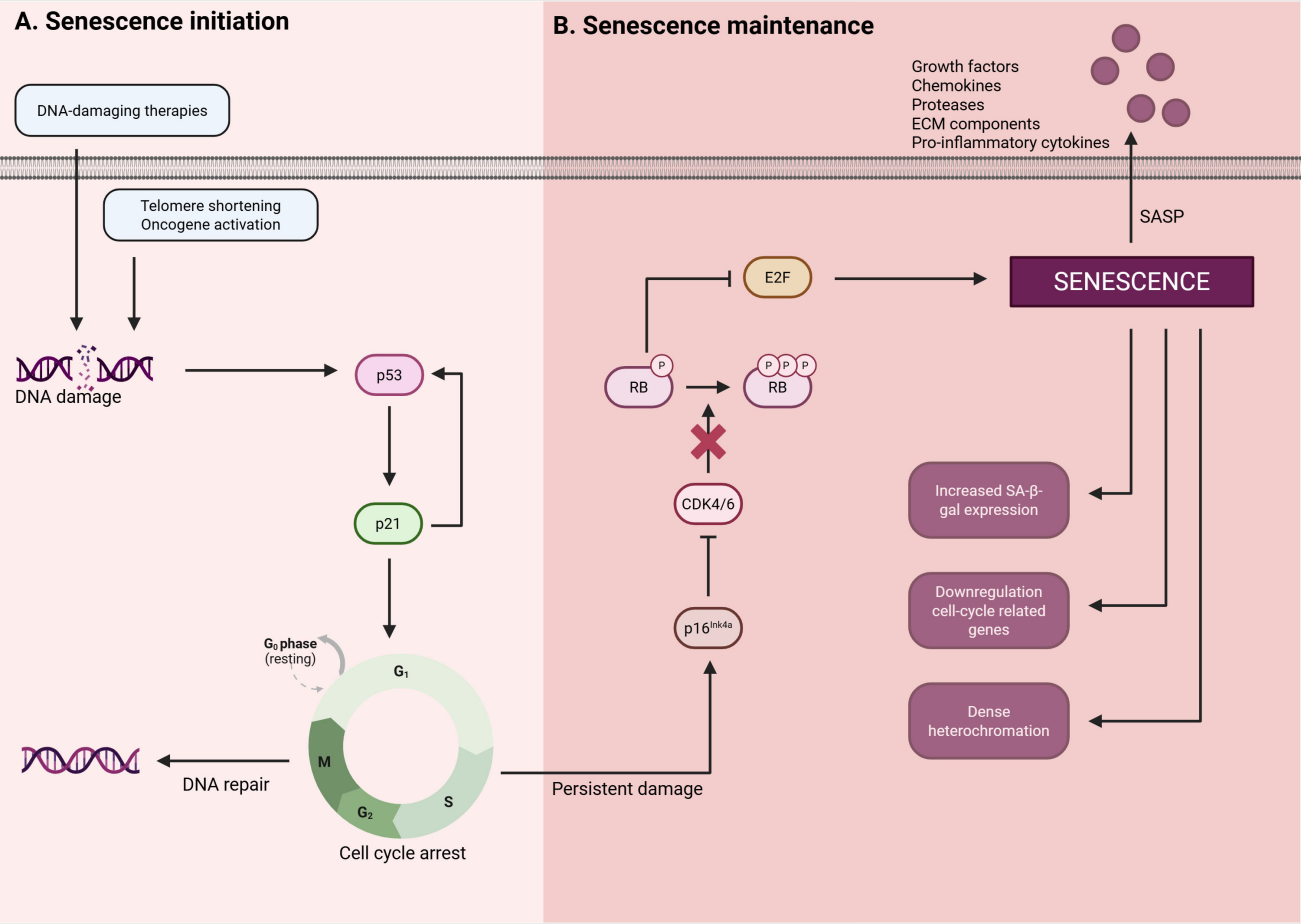
Senescence pathway. **(A)** Senescence can be initiated in response to intra- and extra-cellular stressors, including telomere shortening, oncogene activation, and DNA-damaging therapies. The resulting DNA damage activates p53. Increased p53 expression and transcriptional activity promote induction of downstream targets such as p21, which stabilizes p53 and mediates cell cycle arrest, allowing time for DNA repair. **(B)** Persistent DNA damage activates the p16/RB pathway through upregulation of p16^Inka^. Elevated p16^Inka^ inhibits CDK4/6, preventing RB phosphorylation. Hypophosphorylated RB suppresses E2F transcription factor activity, thereby reinforcing and maintaining long-term senescent growth arrest. Senescent cells exhibit molecular features including heterochromatin foci, SA-β-gal activity, downregulation of cell cycle related genes, and acquisition of a SASP. p53, tumor protein p53; p21, cyclin-dependent kinase inhibitor 1A; p16^Ink4a^, cyclin-dependent kinase inhibitor 2A; CDK4/6, cyclin-dependent kinases 4 and 6; Rb, retinoblastoma protein; E2F, E2 promoter-binding factor; SASP, senescence-associated secretory phenotype; SA-β-gal, senescence-associated β-galactosidase; ECM, extracellular matrix.

Mechanistically, senescence is reinforced through two major signaling axes: the p53/p21 and p16/retinoblastoma (RB) pathways. Upon entry into senescence, both the expression level and transcriptional activity of p53 increase and are sustained through multiple positive feedback loops (96). Elevated p53 promotes transcription of downstream targets such as p21, which maintain p53 stabilization and enforces an initial growth arrest (**Figure 5A**) (97, 98). This p53/p21 axis primarily initiates cell cycle arrest, during which DNA damage may be repaired, potentially allowing cells to re-enter the cell cycle. However, if the arrest persists, the p16/RB pathway becomes activated through upregulation of p16^Ink4a^ (35). Increased p16^Ink4a^ expression inhibits CDK4/6, preventing phosphorylation of RB. In its hypophosphorylated state, RB suppresses E2F transcription factor activity, thereby locking cells into long-term proliferative arrest and stabilizing the senescent phenotype (**Figure 5B**) (99).

#### 3.4.2 Senescence responses in TRT

Insights from EBRT provide a useful framework for understanding how senescence emerges after irradiation, as temporal features of senescence in relation to absorbed dose and dose rate have been studied extensively in primary cell lines exposed to external radiation. In fibroblasts, senescence increases in a dose-dependent manner and becomes apparent after the apoptotic peak declines (100). In HUVECS cultures, higher dose rates accelerate the onset of senescence, although the final proportion of senescent cells was similar across dose rates (101). These findings indicate that the kinetics of damage accumulation, rather than total absorbed dose alone, influences when senescence is established. Because dose rate in TRT fluctuates dynamically due to radionuclide decay and clearance, such kinetics may contribute to heterogeneous senescence responses across radionuclides, tissues and tumor contexts.

Contrarily, continuous radiation given by TRT over hours to several days exposes cells to recurrent cycles of partial repair and renewed damage, creating a sustained stress environment that is likely distinct from pulsed high dose rate delivery in EBRT. Evidence from β^-^-emitter studies indicate a delayed response showed by the gradual emergence of senescence markers. In prostate cancer xenografts, chronic β^-^-irradiation resulted in increased SA-β-gal staining and reduced Ki-67 expression (widely used marker for cell proliferation (102)), emerging at day 4 and peaking at day 14 after treatment, indicating impaired proliferative capacity (103). By 30 days post-treatment, SA-β-gal levels declined while Ki-67 expression increased, consistent with regrowth of non-senescent cells outcompeting the senescent population. Similarly, dose-dependent increases in senescence markers were observed following ^177^Lu-DOTA-TATE treatment, both *in vitro* and *ex vivo*, with regions retaining lutetium-177 showing stronger senescence signatures (77). Here, senescence emerged around day 5, shortly after the apoptotic peak, and continued to accumulate thereafter. Senescent cells also displayed persistent γH2AX foci, consistent with sustained DNA damage signaling.

Importantly, therapy−induced senescence is not restricted to β^-^−emitters, but appears to be a shared outcome across radiopharmaceutical modalities. Emerging evidence indicates that also α−emitters such as radium-223, astatine-211 and actinium-225 can induce senescence following targeted irradiation, reflecting a broader paradigm of radiation−induced cellular reprogramming in surviving tumor cells (104). However, while senescence induction itself appears to be a common response, the qualitative features of the associated SASP remain far less well defined in the context of TRT. In particular, α−particle therapy may exhibit distinctive biological effects. Treatment with [^211^At]-AITM induced G2/M arrest within 24 hours, followed by persistent DNA damage and establishment of a stable senescent phenotype (105). This occurred even in p53-mutant tumors, indicating that prolonged DDR signaling can drive senescence independently of p53. Notably, this senescence state was accompanied by a distinctly reprogrammed SASP with anti-angiogenic and anti-metastatic features, suggesting that α-particle-induced senescence and its modified SASP may act together to constrain tumor growth. While intriguing, it remains unclear whether this SASP profile is specific to α−particle exposure, reflects high−LET radiation more generally, or represents a context−dependent and potentially isolated observation.

Together, these findings demonstrate that senescence can arise as a significant cellular outcome of TRT when cells experience prolonged, sublethal, and sustained stress. However, the current limited mechanistic insights in its initiation, stability, and SASP output underscore the need for TRT-specific studies.

### 3.5 Other responses to TRT

Beyond apoptosis and senescence, which represent major cell-intrinsic responses to TRT, continuous internal irradiation also triggers a broader spectrum of cell-intrinsic, cell-extrinsic, and systemic effects. The low-to-intermediate dose rate exposure characteristic of TRT creates a prolonged and heterogenous stress environment capable of activating additional regulated and unregulated cell death pathways, altering metabolic homeostasis, and reshaping the tumor microenvironment. This includes intercellular signaling processes such as the radiation-induced bystander effect, in which non-irradiated cell respond to signals from irradiated neighbors (12, 106). Moreover, stressed or dying cells release immunogenic signals, allowing TRT to extend its influence beyond irradiated cells and modulate the innate and adaptive immune system. Collectively, these processes highlight that TRT elicits a multifaceted biological response that cannot be captured by apoptosis or senescence alone.

#### 3.5.1 Necrosis

Necrosis is classically defined as an unprogrammed mode of cell death triggered by severe and irreparable injury. It is characterized by organelle swelling, resulting in the loss of plasma membrane integrity and the uncontrolled release of intracellular contents into the surrounding tissue. These released components, known as damage-associated molecular patterns (DAMPs), activate surrounding immune cells and elicit a strong pro-inflammatory response, in contrast to the immunologically silent nature of apoptosis (107). In radiation contexts, necrosis typically develops when the extent of injury exceeds the cell’s ability to engage regulated responses such as apoptosis or senescence. In EBRT, radiation-induced necrosis is a major dose-limiting toxicity, particularly in the brain. Its incidence strongly depends on total dose and fraction number, and it generally manifests as a late complication, often emerging months after treatment has concluded. Although the precise mechanisms remain debated, radiation-induced vascular injury and subsequent tissue ischemia, as well as direct parenchymal damage, are widely implicated (108).

For TRT, the contribution of necrosis is less well defined. The protracted, low dose rate exposure characteristic of TRT generally allows cells to activate regulated responses such as apoptosis, rather than undergoing the unregulated loss of membrane integrity that characterizes necrosis (109). Nevertheless, histological analyses of treated tumors occasionally report necrotic cores following TRT (110–113), although it remains unclear whether these arise directly from radiation damage or from secondary factors such as ischemia and metabolic stress in poorly perfused tumor regions. High-LET α-emitters may further increase the likelihood of necrotic injury in regions receiving very high local energy deposition.

The clinical relevance of necrosis in TRT is currently under investigation. An ongoing clinical trial (NCT07054346) is evaluating necrotic responses following ^177^Lu-PSMA-617 and ^225^Ac-PSMA-617, with a focus on how necrosis is distributed within tumors and how this spatial pattern differs between β^-^- and α-emitting therapies. The study also evaluates whether the degree of necrosis correlates with absorbed dose and treatment response markers. Together, these efforts reflect growing interest in determining when necrosis emerges during TRT and whether it contributes to therapeutic outcome.

#### 3.5.2 Necroptosis

While necrosis represents an unprogrammed response to overwhelming injury, necroptosis is a regulated cell death pathway that produces a similar necrotic morphology when apoptosis is unavailable or actively suppressed. Necroptosis is most commonly initiated by death-receptor signaling, particularly through TNF receptor 1 (TNFR1). Under normal conditions, TNFR1 activation recruits caspase-8, which drives apoptosis and simultaneously suppresses necroptosis. However, when caspase-8 activity is blocked or insufficient, signaling is redirected toward the formation of the necrosome, a multiprotein complex centered on receptor-interacting protein kinases RIPK1 and RIPK3 (107). Within the necrosome, RIPK3 phosphorylates mixed-lineage kinase domain-like protein (MLKL), which oligomerizes and translocates to the plasma membrane. Activated MLKL disrupts membrane organization and forms protein pores, causing ion influx, cell swelling, and eventual rupture (114). This rupture results in the release of DAMPs and triggers a strongly pro-inflammatory form of cell death (115).

Necroptosis is increasingly recognized as a backup mechanism that ensures cell elimination when apoptotic pathways fail. This is particularly relevant in cancer, where apoptosis resistance is common, and in contexts where caspase activity is compromised. IR can promote necroptosis either by activating RIPK1/RIPK3 signaling or by impairing caspase-8 (116, 117). Radiation-induced caspase-8 suppression is thought to involve ROS-driven destabilization of the caspase-8 complex and shifts in TNF-receptor signaling that favor necrosome formation, with such effect especially evident at higher doses (118). Although the contribution of necroptosis to TRT remains less well defined, the pro-inflammatory nature of this pathway and activation under caspase-deficient conditions make it a potential contributor to TRT-response, especially in apoptosis-resistant tumors such as with dysfunctional p53, or in regions receiving high local absorbed doses.

#### 3.5.3 Ferroptosis

Ferroptosis is an iron-dependent, regulated form of cell death driven by the accumulation of lipid peroxides in cellular membranes. Unlike apoptosis or necroptosis, ferroptosis is defined by oxidative damage to polyunsaturated phospholipids and a collapse of the glutathione-GPX4 antioxidant system. When intracellular glutathione (GSH) becomes depleted or GPX4 activity is impaired, lipid hydroperoxides cannot be detoxified and instead undergo iron-catalyzed oxidation, resulting in compromised membrane integrity (119). Multiple upstream stressors can converge in this loss of antioxidant protection. Limiting cystine import, directly inhibiting GPX4, depleting auxiliary antioxidant systems, or increasing the availability of redox-active iron can all shift the intracellular environment toward lipid peroxidation (120). Morphologically, ferroptotic cells exhibit shrunken mitochondria with condensed membranes and reduced cristae, while the plasma membrane remains intact (119).

Ferroptosis has gained attention as a potential contributor to radiation-induced tumor cell death. IR generates ROS which can deplete GSH and thereby weaken GPX4-dependent antioxidant defenses (119). Radiation can also increase the abundance of peroxidation-prone lipids and activate stress-response pathways that sensitize cells to ferroptosis (121). In addition to these direct oxidative effects, radiation-induced DNA damage activates ATM and p53 signaling, which can contribute to GSH depletion and promote susceptibility to lipid peroxidation (119).

Similar mechanisms are being explored in the context of TRT. Low-LET β^-^-emitters such as iodine-131 and lutetium-177 rely heavily on ROS production, creating a redox environment that is likely sensitive to ferroptosis. In FAP-targeted TRT using iodine-131, ferroptosis has been reported in cancer-associated fibroblasts through disruption of iron homeostasis, increased ROS and labile iron levels, and suppression of GPX4 (122). Similarly, treatment of tumor cells with iodine-131-based TRT resulted in downregulation of GPX4 and enhanced oxidative stress. Together, these findings support the idea that depletion of a GPX4 essential cofactor such as GSH possibly represents a key upstream event that permits ferroptotic signaling in TRT.

Despite these indications, definitive evidence that ferroptosis constitutes a major mode of TRT-induced lethality is still limited. Additional studies measuring ferroptosis-specific markers (e.g. ACSL4 induction, lipid peroxide profiling, ferroptosis rescue by lipophilic antioxidants or iron chelators) will be essential to establish the extent to which ferroptosis contributes to TRT response across radionuclides, tumor types, and dose rate conditions.

#### 3.5.4 Autophagy

Autophagy is a process in which cells degrade and recycle cytoplasmic components through lysosomal digestion. Under basal conditions, it maintains metabolic homeostasis by removing damaged organelles and misfolded proteins. In stress context, including nutrient deprivation, hypoxia, and radiation exposure, autophagy is rapidly upregulated as a survival mechanism that preserves energy balance and limits oxidative damage. Morphologically, it is characterized by the formation of double-membrane autophagosomes that consume cytoplasmic material and subsequently fuse with lysosomes for degradation (107).

Induction of autophagy in response to IR is mainly regulated via the PI3K/Akt/mTOR pathway. Autophagy may serve a cytoprotective role by removing damaged mitochondria and reducing ROS accumulation, thereby enhancing radioresistance. However, when autophagy becomes excessive or lysosomal processing is impaired, it can shift toward autophagy-dependent cell death (119). In X-ray irradiated fibroblasts, autophagy functions as a transient intermediate response, peaking around 48h post-irradiation after early apoptosis but before establishment of senescence. This timing suggests that autophagy may help cells manage persistent stress before committing to longer-term fates (100).

Autophagy has also been observed following TRT. Treatment with β^-^-emitters induces autophagy with a temporal pattern similar to EBRT, although the overall response is weaker. This likely reflects the lower dose rate of TRT, which produces more moderate and fluctuating ROS levels (28). In ^131^I-FAP-2286 therapy, autophagy is activated in a dose-dependent manner, and pharmacological inhibition of autophagy enhances therapeutic efficacy, indicating that autophagy can function as a protective response in this setting (123). Conversely, after α-particle TRT, autophagy has been implicated in promoting tumor cell death, as inhibition of autophagy reduced treatment efficacy (124). These findings highlight that autophagy is a context-dependent response to TRT, capable of supporting either cell survival or cell death depending on radiation type, dose rate, and the cell’s ability to maintain metabolic and redox balance.

#### 3.5.5 Immune response

Beyond the induction of local cell death, TRT also modulates the immune system. The immunologic consequences of radiation have been extensively characterized in EBRT studies, where irradiation can induce immunogenic cell death (ICD). This process is marked by the release or surface exposure of DAMPs (such as ATP, HMGB1 and calreticulin), together with the accumulation of cytosolic DNA that activates the cGAS-STING pathway (125, 126). STING signaling drives type I interferon production and pro-inflammatory remodeling of the tumor microenvironment. In parallel, radiation-induced antigen release promotes dendritic-cell recruitment and antigen presentation, ultimately supporting the expansion of CD8^+^ T cells and engagement of the adaptive immune response. However, activation of the same cGAS-STING-IFN1 axis can also promote immunosuppressive features, including recruitment of regulatory T cells (Tregs) and myeloid-derived suppressor cells (MDSCs), as well as upregulation of PD-L1 (127).

The shift between immunostimulatory and immunosuppressive effects after irradiation may be dose-dependent, reflected by dose-responsive changes in several immune markers after EBRT (128, 129). Similar dual effects have been observed in TRT. Both β^-^- and α-emitting radionuclides can induce hallmarks of ICD, including DAMP expression (130, 131) and activation of the cGAS-STING-IFN pathway (132, 133). Several studies report enhanced infiltration of immunostimulatory immune cells, such as CD4^+^ and CD8^+^ T cells (130, 133, 134) and antigen-presenting cells (135), alongside reduced infiltration of immunosuppressive populations including Tregs and tumor-associated macrophages (136, 137). Notably, CD8^+^ T-cell activation correlates with the absorbed dose in TRT, suggesting that a minimum immunogenic dose may be required to convert immunologically cold tumors into more inflamed, T cell-responsive lesions (138). In line with these observations, TRT is increasingly being investigated in combination with immunotherapeutic strategies such as immune checkpoint inhibitors, leveraging radiation-induced immune activation to enhance treatment response. However, varying outcomes have been reported depending on tumor type and treatment (127).

Conversely, TRT can also elicit immunosuppressive signaling. β^-^-emitter studies have shown increased infiltration of PD-L1-positive immune cells into the tumor microenvironment (134, 139), and tumor biopsies from patients treated with α-emitting radionuclides demonstrate PD-L1 upregulation (140). In some models, decreased infiltration of CD4^+^ and CD8^+^ T cells has been observed following TRT (136), underscoring that immune modulation is context-dependent and influenced by radionuclide properties, dose rate, dose distribution and tumor biology.

## 4 Current limitations & future directions

Despite growing interest in the cellular effects of TRT, the current radiobiological evidence base remains fragmented. Mechanistic studies are still limited in number, and those that exist often differ substantially in methodology, experimental models, and biological endpoints. For example, direct comparisons between high- and low-LET TRT are scarce, and apoptosis and senescence are rarely evaluated in parallel, making it difficult to determine how different cell death pathways interact or compete during continuous irradiation and among different radiation types. A further challenge is the inconsistency in timing of post-treatment analyses. Because TRT delivers radiation gradually over extended periods, the choice of readout time strongly influences the observed phenotype. Together, these factors hinder the ability to draw firm conclusions about TRT-specific biology and limit the interpretability of published findings. In addition, incomplete reporting of key radiopharmaceutical characteristics (e.g. activity concentration, molar activity, internalization and retention kinetics, and intracellular localization) makes it difficult to link biological outcomes to tracer behavior across studies, as highlighted in the schematic summary reported in **Table 1** (141).

**Table 1:** Schematic summary of experimental analysis of radiation-induced apoptosis and senescence discussed in this review.

Response	Study model	Treatment	Absorbed dose	Administered activity	Amount of radiopharmaceutical	Dose rate	Detection method	Readout time after treatment start	Reference
Apoptosis	CAPAN-2; A549 cell lines	^213^Bi-DOTATOC; ^177^Lu-DOTATOC	0.35-3.06 Gy	37 kBq	Not available	Not available	Detection of mono- and oligo-nucleosomes that are released upon apoptosis	Day 1, 2, 4	(25)
Senescence	LNCaP-FGC xenografts	^177^Lu-hu11B6	23–124 Gy	10.2-42.3 MBq	20-30 μg	Not available	Expression of SA-β-gal and Ki-67 (proliferation marker)	Day 4,9,14,30	(103)
Senescence	MDA-MB231; MIA PaCa2; A2058; PANC1 cell lines and xenografts	^211^At-AITM	3.79-4.62 Gy/MBq	18.5 kBq/mL *(in vitro)*; 2.96 MBq (*in vivo)*	Not available	Not available	Cell cycle analysis; SA-β-gal expression; cell morphology	Day 1,2,3,7	(105)
Senescence	Human dermal fibroblasts	X-rays	1–10 Gy	N.A.	N.A.	0.85 Gy/min	Expression of SA-β-gal and Ki-67 (proliferation marker)	Day 1,2,3	(100)
Apoptosis; Senescence	CA20948 cell-line and xenograft	^177^Lu-DOTA-TATE	*Not available*	0.05-2.5 MBq/mL *(in vitro)*; 5–30 MBq *(in vivo)*	50 MBq/nmol (*in vitro)*; 86 MBq/nmol (=60 MBq/μg) (*in vivo*)	Not available	Annexin V (apoptosis marker); SA-β-gal expression	Day 1,2,3,4,5,6,7,8,9,10,11	(77)
Apoptosis	HBL; MM162; COLO-677; EJM; MIA-PACA-2; HT-29 cell lines	^177^Lu-DOTA-TATE; X-rays	4-4.7 Gy (^177^Lu-DOTA-TATE); 2 Gy (X-rays)	5 MBq (^177^Lu-DOTA-TATE)	Not available	4 Gy/min (X-rays)	Annexin V (apoptosis marker)	Day 3,10	(28)
Apoptosis	GOT1 xenograft	^177^Lu-octreotate	0.73-6.4 Gy	15 MBq	Not available	Not available	Expression of pro-apoptotic genes *(APOE, BAX, GADD45A, PBK, TNFRSF10B, NGFRAP1)*	Day 1,3,7,41	(78)
Apoptosis	GOT1 xenograft	^177^Lu-octreotate	0.45-4.0 Gy	15 MBq	26 MBq/μg	Not available	Expression of pro- *(APOE, BAX)* and anti-apoptotic *(ADORA2A, BNIP3, BNIP3L, HSPB1)* genes	Day 1,3,7,41	(79)
Apoptosis	GOT1 xenograft	^177^Lu-octreotate	0.27-0.51 Gy/MBq	15–30 MBq	26 MBq/μg	Not available	Transcriptomic profile	Day 1, 3, 7, 41,	(80)
Apoptosis	U2OS; Ca20948 cell lines	^177^Lu-DOTA-TATE	*Not available*	5 MBq	53 MBq/nmol	Not available	Cytochrome C release	Day 1,2,3	(38)
Senescence	HUVECS	^137^Cs	0.24-6.9 Gy	N.A.	N.A.	1.4; 2.5; 4.1 mGy/h	SA-β-gal expression	Week 1,3,6,10,12,15	(101)
Apoptosis	Raji cell line	^177^Lu-anti-CD20	2.61-4.8 Gy	1.85 MBq/mL	1.85 MBq/0.25 mg	Not available	Annexin V (apoptosis marker)	Day 1,2,4	(81)

This table highlights representative studies discussed in this review and is not intended to provide an exhaustive overview of all radiation-induced response pathways or TRT studies.

A major step toward improving the interpretability is the integration of standardized dosimetry into biological experiments. Unlike EBRT, the absorbed dose in TRT varies widely across tumor types, radionuclides, time points and uptake heterogeneity (142). Without quantifying absorbed dose, accurate dose-effect relationships cannot be established. However, absorbed dose alone cannot capture the radiobiological complexity of TRT. Dose rate changes continuously during TRT and influences the temporal dynamics in DNA damage induction, thereby shaping downstream cellular responses. Indeed, some biological end points correlate more strongly with (maximum) dose rate then with absorbed dose (138). *In vivo*, tumor volume adds another layer of uncertainty, as changes in volume affect both local activity per gram and S-values (mean absorbed dose to target tissue per unit activity). Moreover, treatment-induced tumor shrinkage during prolonged TRT exposure may dynamically alter both absorbed dose distributions and tumor oxygenation over time, potentially modifying radiosensitivity through reoxygenation of previously hypoxic regions (143). Under these conditions, the spatial position of decay becomes increasingly important, and average absorbed dose may not reliably predict response. Future studies should therefore integrate multiple dose descriptors, including dose rate, dose rate gradients, microdosimetric spectra, subcellular dose distribution, and cumulative exposure time, to better reflect the biology of continuous irradiation. Such integrated approaches will support the development of quantitative radiobiological models that relate dose delivery to biological effect and help optimize clinical schedules to improve therapeutic outcomes.

A more comprehensive dosimetric description, combined with well-designed biological studies that clearly define TRT-induced responses or cell fate outcomes, will also improve the interpretation of the BED and help translate preclinical findings into opportunities for clinical improvement. Understanding how cells adapt under prolonged irradiation, including the balance between repair, growth arrest, and cell death pathways, may inform optimized scheduling strategies such as fractionation or priming approaches to prevent resistance (19, 78). This is particularly relevant when combining TRT with DNA repair inhibitors or agents that modulate the cell cycle, as it may guide the timing of such treatments to maximize radiosensitization. In this context, targeted inhibition of DDR pathways, including ATR, CHK1, and WEE1 have emerged as promising radiosensitization strategies in EBRT, but remain largely unexplored in TRT (144).

Finally, the contribution of alternative cellular responses, as well as the immunological consequences of TRT, remain underexplored. The potential for TRT to induce immunogenic cell death offers opportunities to enhance antitumor efficacy by combination with immune checkpoint blockade, which can prevent tumor-mediated suppression of T-cell activity and amplify TRT-induced immune responses, particularly in immunologically “cold” tumors (145). However, the dual capacity of TRT to elicit both immunostimulatory and immunosuppressive signaling underscores the need to identify parameters, such as LET, dose rate, dose distribution, and tumor-specific microenvironmental features, that determine the direction of immune polarization.

Overall, advancing TRT radiobiology requires an integrated framework that links physical dose descriptors, radionuclide behavior, DNA-damage dynamics, cell fate decisions, adaptive responses, and immune modulation. Improved dosimetric precision can guide treatment scheduling and personalization, while deeper mechanistic understanding will support rational combination strategies to enhance tumor sensitivity and overcome resistance. In this way, radiobiological insights can be fully leveraged to optimize TRT and translate biological knowledge into clinical benefit. Ultimately, although this review focuses on tumor-intrinsic responses, a comprehensive understanding of TRT will require integration with systemic factors, including radiopharmaceutical distribution, circulation-associated irradiation, radiochemical stability, and associated normal tissue effects, which together shape dose distribution and the balance between tumor control and off-target effects, to enable safe and effective clinical translation.

## 5 Conclusion

Altogether, current evidence on TRT radiobiology indicates that its cellular effects cannot be fully understood by concepts derived from EBRT. The continuous, low dose rate exposure characteristic of TRT changes how DNA damage is processed, how checkpoints are engaged, and how cells progress toward apoptosis, senescence, or alternative death pathways. These dynamics create biological responses that differ not only in magnitude but also in timing and quality from those observed under acute, high dose rate irradiation. A better understanding of parameters that shape these responses will be essential for translation of biological mechanisms into therapeutic improvement. Integrating physical dose descriptors with mechanistic insight into DNA damage signaling, cell-fate decisions, adaptive responses, and immune modulation will be critical for identifying vulnerabilities unique to continuous irradiation. Continued refinement of TRT radiobiology will ultimately support more rational treatment design that enhances tumor sensitivity while minimizing long-term adverse effects, shaping the next generation of TRT.
